# Knowledge, Attitudes and Perceptions Among Non-Blood Donor Female Health Care Professionals

**DOI:** 10.5539/gjhs.v8n4p203

**Published:** 2015-08-18

**Authors:** Muhammad Bilal, Abdul Haseeb, Ibrahim Zahid, Sehan Siraj Lashkerwala, Fawad Saeeduddin, Muhammad Saad, Mohammad Hussham Arshad, Manpreet Moorpani, Midhat Zafar Khan, Ahsan Tariq, Haya Habib, Tehrema Islam, Rohan Advani

**Affiliations:** 1 Dow University of Health Sciences, Karachi, Pakistan; 2 Jinnah Sindh Medical University, Karachi, Pakistan; 3 Department of Pulmonology, Jinnah Postgraduate Medical Center, Karachi, Pakistan; 4 Aga Khan University Hospital, Karachi, Pakistan; 5 The Lyceum, Karachi, Pakistan; 6 Karachi Grammar School, Karachi, Pakistan

**Keywords:** non-blood donors, female health care professionals, knowledge, perceptions, tertiary care hospitals

## Abstract

**Introduction:**

Blood donation is necessary in order to maintain an adequate supply of blood to patients who are suffering from any kind of disease or trauma, which requires them to have blood transfusion. Female non-blood donors are generally low in number. Therefore, this research was carried out to assess the main reasons behind the lack of blood donations made by females, and their knowledge, attitude and perceptions towards voluntary blood donation.

**Methodology:**

A cross-sectional study was conducted on 664 female health professionals, who were selected by non-probability convenience sampling from two tertiary care hospitals. A pretested questionnaire was presented to the sample population, and the data was entered and analyzed on SPSS (V17).

**Results:**

94.6 % were aware with the fact that blood is screened for AIDS, Hepatitis B and C before transfusion. Moreover, 83.7% said that they will only donate blood if a family, relative or friend would need it and similarly 83.4% suggested that they would donate blood if blood donation camps are arranged in hospital premises. 81.8 % thought that blood donors can contract Hepatitis B after donation whereas only 29.5% did not blood due already blood loss in menstrual cycle.

**Conclusion:**

The participants had adequate knowledge about the benefits of blood donation. The most important reason identified for not donating blood is the lack of facilities within the workplace or lack of approach by responsible authorities. The results of the study may help in minimizing the misconceptions of the participants about blood transfusion, which would increase their contribution towards blood donation.

## 1. Introduction

Blood donation refers to the process of collecting, testing, preparing and storing blood and its components (http://www.mayoclinic.org/). The purpose of the blood collection and distribution system is to help ensure an adequate supply of blood for accident victims, people needing surgery, people suffering from liver disease and those suffering from certain diseases like anemia, thalassemia, hemophilia, as well as for medical research (http://www.mayoclinic.org/). For a country to maintain a basic, self-sufficient supply, it is roughly estimated that 2-2.5% of the population must give blood regularly [World Health Organization, Blood safety: Key global fact and figures in 2011. (Online) (Cited 2013 July 1). Available from URL: http://www.who.int/topics/blood_safety/en/]. According to WHO, about 10 000 blood centers in 168 countries report collecting a total of 83 million donations. Women constitute to only 30% of global blood donations and in 20 of the 111 reporting countries, less than 10% donations are given by female donors (Bani & Giussani, 2010). Men have given blood more often than women. Consequently, the blood transfusion services are facing a major challenge to meet the increasing demand of the blood.

In Pakistan more than 1.5 million pints of blood are collected each year. Among them about 65% is from replacement donors, 25% from volunteer donors and only about 10% from professional donors (Asif, Kokhar, & Ilahi, 2004). Unfortunately, Pakistan is one of those countries where blood donation rate is less than 1%, as stated in a newspaper published in 2012. According to estimates, over 70% blood donations in Pakistan are replacement or paid for donations while unscreened blood transfusion is done in over 50% of the cases. Reports by WHO suggest that over 90% of total blood transfused in Pakistan is donated by the friends and relatives of patients. However, despite efforts to control the practice, around 10%–20% of the blood supply is still donated by professional donors. The average number of blood donations per 1,000 populations is 12 times higher in high income countries than in low-income countries [World Health Organization (WHO) Blood donation factsheet 2009]. The factors responsible for such low blood collection in Pakistan include lack of education and awareness about the need of safe blood in the community, importance of voluntary unpaid blood transfusion (VUBD) and high prevalence of hepatitis B, C, HIV/AIDS and anemia.

Many reports in the past have highlighted the insufficient knowledge and numerous misconceptions of people about blood donation (Kowsalya, Vijakumar, & Chidambaram, 2013). Keeping in view the significance of blood donation and the decreasing fraction of transfused blood over recent years, this research was conducted to evaluate the reasons as to why sufficient blood donations are not made. Since female donors are much less than male donors, the research focused on females and their attitude towards not donating blood. Therefore, the main objective of the study was to assess the knowledge, attitudes and perceptions among non-blood donor female health professionals of tertiary care hospitals in Karachi towards voluntary blood donation.

## 2. Methodology

A descriptive, cross sectional survey was conducted in two tertiary care hospitals of Karachi over a period of 2 weeks in March 2015. Assuming the knowledge of blood donation among non-blood donor female health care professionals to be 50% (taking 99% level of significance and 1% confidence limit), the sample size calculated was 664. Researchers, after explaining the purpose of study and taking written consent, presented a self-administered questionnaire to the sample population using the non-probability convenience sampling method. Health care professionals who did not consent to participate were excluded from the study. The study was performed after the approval from the Institutional Review Board of the Dow University of Health Sciences.

This multicenter study was conducted at Civil hospital and Jinnah post graduate medical center, Karachi. Questionnaires were distributed among the target population after a pilot study which included female nurses, interns, residents and consultants who had never donated blood in their life. Exclusion criteria included all male health care professionals, female health care professionals who had donated blood at least once in their lifetime, females suffering from hemophilia and thalassemia; and females on blood thinners. The questionnaire was divided into 3 major parts. The first part consists of the demographic characteristics (for e.g. age, sex, income) of the female health care professional. Second part was related to the knowledge of target population regarding blood donation. Question investigating perceptions of not donating blood concluded the third part of the questionnaire.

Data was collected and entered on SPSS (V17) software. The same software was used for data management and analysis. Standard deviation and mean were calculated for the continuous variables like age. Frequencies and percentages were calculated for qualitative variables like gender, level of education, marital status etc. Chi-square was used to deduce the association between the knowledge and perceptions of blood donation of different medical health professionals. P value less than 0.05 was taken as significant.

## 3. Results

A total of N= 664 participants fulfilling the inclusion criteria were recruited in the survey. Out of them 39.76% (N= 264) were Interns, 23.34% (N= 155) were residents, 4.37% (N=29) were consultants and 32.53% (N=216) were nurses. The mean age of participants was 26.7±2.5. Seventy one percent (N=469) had level of education of bachelors, 59.64% (N=396) participants had income of 10,000-25,000 rupees, 69.28% (N=460) of study population was unmarried and consequently 78.31% of the participants (N=520) were childless. Table 1 depicts demographics of female health professionals

**Table 1:** Demographics of female health professionals

Demographic Characteristics	n	%
1. Designation		
Intern	264	39.76
Resident	155	23.34
Consultant	29	4.367
Nurse	216	32.53
2. Age (Mean ±SD)	26.7±2.5	-
3. Level Of Education		
Matric	56	8.434
Intermediate	82	12.35
Bachelors	469	70.63
Masters	57	8.584
4. Income (Rupees)		
10,000-25,000	396	59.64
26,000-50,000	187	28.16
> 51,000	81	12.20
5. Marital status		
Married	204	30.72
Unmarried	460	69.28
6. Number of children		
0	520	78.31
1- 2	103	15.51
3 - 4	41	6.175

When non blood donor female participants were questioned about their knowledge and attitude towards blood donation, 94.6% (N=628) knew that blood is screened for AIDS, Hepatitis B and C before transfusion and similar frequency was noted for those who responded that blood can be used in cancer treatment. Eight four percent of the participants (N=555) were aware about the fact that all surgical procedures require blood transfusion and transfused blood can be stored, 91.3% (N=606) said that blood is required in accident and emergency department and 81.8% (N=543) were also aware that blood can be donated during fast. Moreover, 83.7% (N=556) said that they will only donate blood if a family, relative or friend would need it and similarly 83.4% (N=554) suggested that they would donate blood if blood donation camps are arranged in hospital premises. The most prevalent misconception among the respondents was that the blood donor has risk for contracting infections as 81.8% (N=543) said for Hepatitis B, 83.4% (N=554) said for Hepatitis C and 78.7% (N=523) considered HIV to be contracted after donating blood. However, the misconceptions regarding the risk factors associated with blood transfusion were significantly low as only 16.1% (N=107) considered weight gain after blood donation, 20.0% (N=133) said weight loss, 5.4% (N=36) said infertility and only 20% (N=133) thought of having severe fatigue and bruising at injection site. Additionally, when questioned about the conditions in which blood should not be donated, 59.0% (N=392) said alcoholics, 69.4% (N=461) responded communicable disease, 75.9% (N=504) considered that having history of Malaria/Hepatitis and 40.6% (N=270) thought that person having history of allergies should not donate blood. Lastly, only 58.1% (N=386) replied positively with regards to donating blood in future. Table 2 depicts knowledge and attitude of female health professionals.

**Table 2:** Knowledge and attitude of female health professionals

	Intern (n = 264)		Resident (n =155 )		Consultant (n = 29)		Nurse (n =216 )		p-values

	n	%	n	%	n	%	n	%	
Blood is screened for aids, hepatitis b & c before transfusion									<0.001
Yes	260	41.4%	155	24.7%	29	4.6%	184	29.3%	
No	4	11.1%	0	.0%	0	.0%	32	88.9%	
All surgical procedures require blood transfusion									<0.001
Yes	240	43.2%	135	24.3%	28	5.0%	152	27.4%	
No	24	22.0%	20	18.3%	1	.9%	64	58.7%	
Blood can be used in cancer treatment									0.305
Yes	260	41.4%	155	24.7%	29	4.6%	184	29.3%	
No	4	11.1%	0	.0%	0	.0%	32	88.9%	
Blood is required in emergencies									<0.001
Yes	244	40.3%	144	23.8%	28	4.6%	190	31.4%	
No	20	34.5%	11	19.0%	1	1.7%	26	44.8%	
Blood can be stored									<0.001
Yes	240	43.2%	135	24.3%	28	5.0%	152	27.4%	
No	24	22.0%	20	18.3%	1	.9%	64	58.7%	
Blood can be donated while keeping a fast									<0.001
Yes	226	41.6%	140	25.8%	25	4.6%	152	28.0%	
No	38	31.4%	15	12.4%	4	3.3%	64	52.9%	
I will donate blood if a family, relative, or friend needs									<0.001
Yes	230	41.4%	133	23.9%	21	3.8%	172	30.9%	
No	34	31.5%	22	20.4%	8	7.4%	44	40.7%	
I would donate blood if blood donation camp arrange in the hospital premises									<0.001
Yes	232	41.9%	145	26.2%	21	3.8%	156	28.2%	
No	32	29.1%	10	9.1%	8	7.3%	60	54.5%	
Which infectious diseases do you think can be contracted after blood donation?									
a. Hepatitis B									<0.001
Yes	226	41.6%	140	25.8%	25	4.6%	152	28.0%	
No	38	31.4%	15	12.4%	4	3.3%	64	52.9%	
b. Hepatitis C									<0.001
Yes	232	41.9%	145	26.2%	21	3.8%	156	28.2%	
No	32	29.1%	10	9.1%	8	7.3%	60	54.5%	
c. Malaria									<0.001
Yes	90	41.5%	77	35.5%	12	5.5%	38	17.5%	
No	174	38.9%	78	17.4%	17	3.8%	178	39.8%	
d. Aids									<0.001
Yes	228	43.6%	136	26.0%	21	4.0%	138	26.4%	
No	36	25.5%	19	13.5%	8	5.7%	78	55.3%	
e. Tuberculosis									<0.001
Yes	16	17.8%	9	10.0%	7	7.8%	58	64.4%	
No	248	43.2%	146	25.4%	22	3.8%	158	27.5%	
What do you think are the risk factors associated with transfusion?									
a. Weight gain									<0.001
Yes	16	15.0%	11	10.3%	6	5.6%	74	69.2%	
No	248	44.5%	144	25.9%	23	4.1%	142	25.5%	
b. Weight loss and permanent weakness									<0.001
Yes	30	22.6%	17	12.8%	2	1.5%	84	63.2%	
No	234	44.1%	138	26.0%	27	5.1%	132	24.9%	
c. Infertility									
Yes	6	16.7%	4	11.1%	0	.0%	26	72.2%	
No	258	41.1%	151	24.0%	29	4.6%	190	30.3%	
d. Bruising at phlebotomy(injection) site									<0.001
Yes	30	22.6%	17	12.8%	2	1.5%	84	63.2%	
No	234	44.1%	138	26.0%	27	5.1%	132	24.9%	
e. Severe fatigue									<0.001
Yes	30	22.6%	17	12.8%	2	1.5%	84	63.2%	
No	234	44.1%	138	26.0%	27	5.1%	132	24.9%	
Choose the conditions in which blood should not be donated									
a. Alcoholism drugs consumption									0.373
Yes	152	38.8%	98	25.0%	20	5.1%	122	31.1%	
No	112	41.2%	57	21.0%	9	3.3%	94	34.6%	
b. Communicable diseases									<0.001
Yes	194	42.1%	125	27.1%	16	3.5%	126	27.3%	
No	70	34.5%	30	14.8%	13	6.4%	90	44.3%	
c. Having previous history of malaria/hepatitis									<0.001
Yes	224	44.4%	116	23.0%	26	5.2%	138	27.4%	
No	40	25.0%	39	24.4%	3	1.9%	78	48.8%	
d. Having a history of allergies									0.001
Yes	118	43.7%	45	16.7%	7	2.6%	100	37.0%	
No	146	37.1%	110	27.9%	22	5.6%	116	29.4%	
Do you plan to donate blood in future?									
Yes	168	43.5%	87	22.5%	15	3.9%	116	30.1%	
No	24	22.0%	27	24.8%	4	3.7%	54	49.5%	
Don’t know	72	42.6%	41	24.3%	10	5.9%	46	27.2%	

When enquired about the perceptions and reasons for not donating blood, 29.5% (N=196) did not blood due to blood loss through menstrual cycle, 36.7% (N=250) had no time for going to blood bank, 13% (N=86) considered female blood as impure and it may harm the recipient, 34.5% (N=229) did not trust the blood banks, 31.5% (N=209) had fear of needle prick and 29.5 (N=196) had apprehension of syncope and becoming weak after donation. Furthermore, 31.3% (N=208) did not consider them strong enough to give blood as men, 47% (N=312) said that they were never asked to donate, 20.1% (N=133) did not considered their information to be sufficient regarding donation. However, 15.4% (N=102) had fear of too much blood to be withdrawn, 32.0% (N=212) considered them to be not eligible medically and only 17.6% (N=117) considered process to be long and boring. Table 3 depicts perceptions and reasons associated among non-blood donor female health professionals.

**Table 3:** Perceptions and reasons associated among non-blood donor female health professionals

	Intern (n = 264)		Resident (n =155 )		Consultant (n = 29)		Nurse (n =216 )		P-Values

	n	%	n	%	n	%	n	%	
1. You do not prefer donating blood due to already losing blood through menstruation (concern of having low blood)									<0.001
Yes	38	19.4%	27	13.8%	3	1.5%	128	65.3%	
No	226	48.3%	128	27.4%	26	5.6%	88	18.8%	
2. No time for going to a blood bank (too busy caring for the family)									0.001
Yes	74	30.3%	60	24.6%	12	4.9%	98	40.2%	
No	190	45.2%	95	22.6%	17	4.0%	118	28.1%	
3. Dirtiness of menstrual blood (Napak/impurity of female blood)									NA
Yes	10	11.6%	8	9.3%	0	.0%	68	79.1%	
No	254	43.9%	147	25.4%	29	5.0%	148	25.6%	
4. The blood may harm the recipient									NA
Yes	16	18.6%	10	11.6%	2	2.3%	58	67.4%	
No	248	42.9%	145	25.1%	27	4.7%	158	27.3%	
5. You don’t trust the blood banks distribution facility and sterilization of equipment									<0.001
Yes	66	28.8%	57	24.9%	6	2.6%	100	43.7%	
No	198	45.5%	98	22.5%	23	5.3%	116	26.7%	
6. A fear of needle prick									<0.001
Yes	60	28.7%	40	19.1%	11	5.3%	98	46.9%	
No	204	44.8%	115	25.3%	18	4.0%	118	25.9%	
7. Apprehension of syncope and becoming weak after donation									<0.001
Yes	60	30.6%	35	17.9%	7	3.6%	94	48.0%	
No	204	43.6%	120	25.6%	22	4.7%	122	26.1%	
8. You do not feel strong enough to give blood as men									<0.001
Yes	62	29.8%	31	14.9%	9	4.3%	106	51.0%	
No	202	44.3%	124	27.2%	20	4.4%	110	24.1%	
9. No-one ever asked for donation and never thought to donate									<0.001
Yes	100	32.1%	63	20.2%	15	4.8%	134	42.9%	
No	164	46.6%	92	26.1%	14	4.0%	82	23.3%	
10. Don’t have enough information									<0.001
Yes	22	16.4%	15	11.2%	1	.7%	96	71.6%	
No	242	45.7%	140	26.4%	28	5.3%	120	22.6%	
11. Fear that they would take too much blood									NA
Yes	18	17.6%	8	7.8%	2	2.0%	74	72.5%	
No	246	43.8%	147	26.2%	27	4.8%	142	25.3%	
12. Not eligible medically									<0.001
Yes	70	32.9%	49	23.0%	2	.9%	92	43.2%	
No	194	43.0%	106	23.5%	27	6.0%	124	27.5%	
13. Process is long and boring									<0.001
Yes	22	18.8%	29	24.8%	0	.0%	66	56.4%	
No	242	44.2%	126	23.0%	29	5.3%	150	27.4%	
NA; Not applicable due to cells have expected count less than 5.									

## 4. Discussion

This descriptive study was conducted to investigate the current information regarding knowledge, attitude and perceptions among non-blood donor female health professionals in leading teaching hospitals of Karachi. The results will be helpful in execution of relevant donor gathering strategies, because this subset of population can contribute significantly to augment health promoting activities in society. This task can be achieved by hospitals blood bank by increasing appropriate distribution of collected blood (Vos, 1998) and also by increasing healthy blood donor recruitment (Gillespie & Hillyer, 2002). The factors leading to decrease blood donation has become an important concern and are studied nowadays worldwide to increase voluntary blood collection (Allen & Butler, 1993).

The demographic features of previous data indicated female donors to be very few in number as compare to male donors as shown by Gillespie (Gillespie & Hillyer, 2002). Moreover, a work by Hollingsworth reported only 1% female donors among donor population (Hollingsworth & Wildman, 2004). Therefore the issue needs to be given due consideration, as female health professionals are the most accessible source of voluntary blood donation.

In general, the knowledge of participants towards benefits of blood donation is sufficient and are well aware with conditions in which blood should not be donated as the study participants belong to medical profession. This is in line with the finding of several university students at India (Giri & Phalke, 2012), Bangladesh (Hosain, Anisuzzaman, & Begum, 1997) and Thailand (Wiwanitkit, 2002). However, Safizadeh (Safizadeh, Pourdamghan, & Mohamadi, 2007) in Iran found that the awareness regarding blood donation was inadequate while a similar study in Pakistan also reported that there is a lack of knowledge and awareness regarding benefits of blood donation (Saeed, Munir, & Shahid, 2011). This is the point to ponder for blood transfusion services that despite sufficient knowledge among female health professionals, a significant number do not donate blood. They must be encouraged in order to increase voluntary blood collection.

Surprisingly, majority of the participants (83%) do not donate blood due to lack of facilities and absence of blood donation camp in hospital premises. The results are congruent with the findings of another study conducted on the undergraduates of a medical college of Pakistan (Ahmed, Zafar, Khan, Anjum, & Siddique, 2014), where majority of the participants reported the same reason. Therefore, steps should be taken to organize blood donor societies and camps within the premises of work place. Furthermore, it should be mandatory by health ministry that health professional should donate blood at least once or twice in a year. This will increase blood donor pool greatly.

The current study further highlighted that among the respondents, the most widespread misconception was that the blood donor has risk for contracting infection like HIV or Hepatitis B and C. This finding has been corroborated by findings of studies carried out in past. A similar study in Pakistan revealed that 10.2% of the participants do not donate blood because of the fear of contraction of diseases after blood donation (Hosain, Anisuzzaman, & Begum, 1997). Furthermore, a study reported misjudgment of acquiring AIDS and hepatitis due to blood donation activities among the French population (Munoz, Bacq, & Mullet, 2002). A study in Nigeria reported that 52.4% students have a similar misconception of acquiring AIDS and hepatitis by blood donation (Olaiya, Alkija, Ajala, & Olatunji, 2004). Additionally, an Iranian study also reported that most of the students (66.6%) had the same misbelief (Safizadeh, Pourdamghan, & Mohamadi, 2007). We suggest that for the success of blood donation campaigns, it is necessary to remove these misconceptions and this could be achieved by the introduction of awareness programs through television, radio and newspaper which are a major source of information in Pakistan.

Additionally, our study also revealed that 47% of participants did not donate because they were not approached by anyone and never thought of donating. This is consistent with the another Saudi study where 45% did not donate for the same reason (Alam, & MasalmehBel, 2004). Around 30% of participants did not donate because of fear of needle prick, apprehension of syncope and becoming weak after donation. This is contradictory with work of Olaiya (Olaiya, Alkija, Ajala, & Olatunji, 2004), where 60% participants did not donate blood because of similar reasons.

The current study shows that the major motivation for donors was to help family or friends, for saving others’ lives and altruism as responded by 85% of responders. This is in line with several studies which have indicated altruism as an important motivating factor among donors in Pakistan (Saeed, Munir, & Shahid, 2011), India (Singh, Pandey, &D’Souza, 2002) and Brazil (Thelma, Goncalez, & Ester, 2008). However this is inconsistent with an American study where participants demanded for incentives like free blood investigations, lottery tickets and souvenirs over altruism (Glynn et al., 2003). Furthermore, very few people (13%) reported female blood as impure and only 30% considered becoming anemic due to already losing blood in menstruation cycle. The frequency of perceptions like women do not feel strong as men, was also considerably low. This is incompatible with other studies conducted on Turkish (Dilsad, Tanriover, Hidiroglu, Gurbuz, & Karavus, 2014) and Pakistani (Mumtaz, Bowen, & Mumtaz, 2012) females which reported higher frequency of these perceptions, indicating reduced frequency of these perceptions among female health professionals.

Statistically significant differences were found with association of knowledge of blood donation and perceptions of not donating blood with medical health professionals. The inadequacy of knowledge along with perception frequency was higher in nurses and least in consultant doctors. The differences could be attributed to medical experience and higher medical knowledge. Therefore awareness programs must be made regular part in training of medical health professionals, so as to diffuse any misconceptions they have regarding voluntary blood donation.

To the best of our knowledge, this is the pioneer study in identifying the knowledge, attitudes and perceptions among non-blood donor female health professionals. The major limitation to our study is that since Karachi is a multicultural city with a broad diversity of traditions, data from two hospitals could not be generalized to other populations of country. Secondly, data on those who did not agree to participate in the survey was not collected and analyzed to exclude the possibility of a sampling bias.

## 5. Conclusion

In general, study participants had sound knowledge of benefits of blood donation and frequency of perceptions and reasons for not donating blood is low in comparison to other study populations. Alarmingly, few misconceptions are still prevalent among female health professionals. The most important reason identified for not donating blood is lack of facilities within the workplace or lack of approach by responsible authorities. So by performing this research, we tend to determine the misconceptions of females, especially the ones who form a part of the working population in the medical field. The results of the study may help to remove the concept of misunderstanding about current issues regarding blood donation and transfusion and may also facilitate to develop donor recruitment strategies within the healthcare premises and educational approaches to enhance blood donors’ participation.
